# Effect of Zinc Sulfate and Zinc Glycine Chelate on Concentrations of Acute Phase Proteins in Chicken Serum and Liver Tissue

**DOI:** 10.1007/s12011-018-1346-6

**Published:** 2018-04-19

**Authors:** Łukasz Jarosz, Agnieszka Marek, Zbigniew Grądzki, Ewa Laskowska, Małgorzata Kwiecień

**Affiliations:** 1 Department of Epizootiology and Clinic of Infectious Diseases, Faculty of Veterinary Medicine, University of Life Sciences in Lublin, Głęboka 30, 20-612 Lublin, Poland; 2 Sub-Department of Preventive Veterinary and Avian Diseases, Institute of Biological Bases of Animal Diseases, Faculty of Veterinary Medicine, University of Life Sciences in Lublin, Głęboka 30, 20-612 Lublin, Poland; 3 Faculty of Biology and Animal Breeding, Institute of Animal Nutrition and Bromatology, Department of Animal Nutrition, University of Life Sciences in Lublin, Akademicka 13, 20-950 Lublin, Poland

**Keywords:** Zinc glycine chelate, Zinc sulfate, Acute phase protein, Chicken

## Abstract

The aim of the study was to determine how inorganic and organic forms of zinc affect the concentrations of C-reactive protein (CRP), serum amyloid A (SAA), alpha-1-acid glycoprotein (α-1-AGP), haptoglobin (Hp), and transferrin (TRF) in the blood and liver tissue of 450 1-day-old Ross 308 chicken. Four experimental groups received one the following: inorganic zinc (ZnSO_4_), a zinc phytase enzyme supplement (ZnSO_4_-F), organic zinc in combination with glycine (Zn-Gly), or organic zinc supplemented with phytase (Zn-Gly-F). The chicken serum and liver homogenates were assayed using an ELISA kit. The results of the study showed statistically significantly higher serum and liver concentration of SAA in the group of birds that received zinc sulfate in comparison to the group of birds receiving zinc in organic form. A statistically significantly higher serum concentration of CRP and α-1-AGP was also noted in the group receiving zinc sulfate as compared to the Zn-Gly group. Comparison of the serum concentration of TRF between the supplemented groups showed a statistically significant increase in this parameter in the Zn-Gly-F group as compared to the ZSO_4_-F group. The increase in the serum concentration of Hp in all groups in comparison to the control may indicate stimulation of local immune mechanisms. The results of this study showed an increase in the concentrations of APPs such as AGP and TRF following the administration of zinc glycine chelates, which may demonstrate their effect on metabolic processes in the liver and on immunocompetent cells that regulate the intensity of the immune response.

## Introduction

The manner of poultry feeding and quality of the feed can have beneficial effects on the health of the birds, mainly by influencing the immune system to ensure the maintenance of homeostasis and protection against infections induced by pathogenic microbes [1–4]. Immune activity in poultry is influenced by micro- and macronutrients, particularly zinc, which is included in poultry diets as feed additives. Zinc has been shown to modulate the body’s immune response [5, 6]. In particular, zinc increases the activation and proliferation of lymphocytes, mainly T and natural killer (NK) cells, and stimulates cellular defense mechanisms [6–8]. The immunomodulatory effect of zinc also results in an increase in the activity of thymocytes, macrophages, and heterophils, as well as increased antibody production, which enhances the potential of the humoral response [9].

The basic components of poultry feed do not contain enough zinc to ensure that these physiological processes proceed correctly [10]. In addition, endogenous losses of zinc are linked to disturbances in intestinal absorption and increased excretion of the element. Zinc deficiency in poultry manifests as impaired resistance and thus increased susceptibility to disease and can lead to debilitation and death [6]. Supplementation of poultry feed with various forms of zinc, usually inorganic forms such as sulfates and oxides, has been recommended [11]. Compound feeds in the poultry industry usually contain a zinc additive in the amount of 100–120 mg Zn/kg [12], despite the fact that the NRC requirement [13] for broiler chickens is 40 mg/kg. Excessive supplementation with inorganic Zn can lead to serious contamination of the environment due to the low utilization of this element. Furthermore, these inorganic compounds have low bioavailability and reduce the reactivity of the immune system by irritating the gastrointestinal mucosa, decreasing the digestibility of other nutrients, and inducing local inflammation [14]. Kidd et al. [15, 16] recommended the use of organic forms of zinc. Zinc chelates, including glycine chelates, have better bioavailability than inorganic forms of zinc [6]. In addition, Kidd et al. [15, 16] have suggested that dietary Zn from organic sources may be absorbed intact and function differently after absorption than Zn from inorganic sources.

The zinc phytate complex forms an insoluble and unabsorbable compound, which is one mechanism reducing zinc availability to animals. Phytase is an enzyme that acts specifically on phytate, breaking it down to release trace elements in a form available to the animal. Phytase is present in low concentrations in the gastrointestinal tract of poultry, but can be added to the diet of broilers to hydrolyze phytate in the gastrointestinal tract, allowing the bound minerals to become available to the animal [17].

The local inflammatory process in the intestines, induced by various exogenous and endogenous factors, including zinc sulfates, leads to immunosuppression, which is conducive to the development of diseases caused by opportunistic microbes [18, 19]. Local inflammation in poultry is accompanied by a systemic response known as the acute phase response (APR) [20–22]. The APR is an early and non-specific systemic reaction of the innate immune system to local or systemic disturbances [22]. This response is primarily modulated by cytokines functioning as cell growth factors and as pro-inflammatory or anti-inflammatory mediators of inflammation [23, 24]. These cytokines are produced and released by activated immune cells. Pro-inflammatory cytokines and chemokines have been shown to activate hepatocyte receptors in the liver, which causes changes in the synthesis and secretion of diverse proteins into the peripheral circulation [25, 26]. These proteins, known as acute phase proteins (APPs), play a profound role in defense mechanisms, by killing infectious microbes, repairing tissue damage, and restoring health [25, 27]. In birds, we can distinguish between positive APPs, which include C-reactive protein (CRP), haptoglobin (Hp), ceruloplasmin (Cp), serum amyloid A (SAA), alpha-1-acid glycoprotein (α-AGP), and fibrinogen [20, 21], and negative APPs, which include albumin and transferrin (TRF) [20, 21]. The concentration of the APPs in birds increases during the development of inflammation [28, 29]. The serum concentration of the APPs in poultry is commonly evaluated to monitor the health of the birds and to determine the intensity of inflammation, as well as in clinical diagnostics [29–34].

Our previous study [6] showed that the use of zinc sulfates as feed additives had no immunomodulatory effects and may have contributed to local inflammation in the intestines, as indicated by high concentrations of pro-inflammatory cytokines, such as IL-2 and tumor necrosis factor alpha (TNF-α). Furthermore, feed supplementation with a zinc glycine chelate (Zn-Gly) together with phytase was shown to exert a similar effect on poultry, but on a smaller scale [6]. However, we could not rule out microdamage to the intestinal epithelium as a consequence of an excessive supply of these compounds. The development of local inflammation in the intestines may lead to a cytokine-dependent reduction in the serum. A decreased serum concentration of zinc is a defense mechanism against its utilization by pathogenic microbes and involves the penetration of zinc into the liver where zinc binds to metalloenzymes, whose functions include the production of new APPs [35]. Synthesis of the APPs in the liver and their release into the peripheral blood are also stimulated by local and systemic production of pro-inflammatory cytokines, such as IL-1, IL-6, and TNF-α [23, 36, 37].

Determination of the concentrations of the APPs in poultry enables early detection of inflammatory processes and is the basis for undertaking medical intervention in order to restore homeostasis [38]. However, the concentration of these proteins depends on numerous endogenous and exogenous factors that are associated with diet [1–3, 39–42]. The available literature contains no information on the effect of feed additives in the form of zinc glycine chelates and zinc sulfates on the concentration of the APPs in poultry. We aimed to determine the effect of inorganic and organic forms of zinc on the concentrations of CRP, SAA, α-AGP, Hp, TRF, and Zn concentration in hepatic tissue of poultry.

## Methods

### Experimental Animals

The experiments were carried out at the Small Animals Teaching and Research Station of the University of Life Sciences in Lublin, Poland. The study was conducted on 450 1-day-old Ross 308 roosters.

The experimental birds were kept in cages in a room with controlled temperature and humidity. The broilers were weighed and randomly placed in battery cages (1 m^2^) with five birds per cage. The metal cages had grates that were replaced as the birds grew. The cages were equipped with nipple drinkers and feeders whose height was continually adjusted to the age of the birds. All cages were in the same room, with electric lighting used 24 h/day until the tenth day of the experiment and 16 h/day from days 10 to 42 of the experiment, according to the lighting scheme for broilers raised on the farm at the Small Animals Teaching and Research Station of the University of Life Sciences in Lublin, Poland. Three days before the chickens were placed in the cages, the floor was heated to 29 °C and the air to 33 °C, with relative humidity of 63%. During the first week of the experiment, the temperature was kept at 33 °C and thereafter was reduced weekly by 2–3 °C until reaching a final temperature of 20–22 °C. Humidity during successive periods of the experiment was as follows: days 1–21: 55–60%; days 22–35: 60–65%; and days 36–42: 65–70%.

The birds were fed ad libitum with compound feeds appropriate for each period of rearing, i.e., starter (S) (days 1–21), grower (G) (days 22–35), and finisher (F) (days 36–42) and had unlimited access to water. The starter mixture was provided to the chickens in crushed form, and the grower and finisher feeds were pelleted. The starter, grower, and finisher feeds were prepared from maize meal, wheat meal, and soybean extraction meal. No coccidiostats or antibiotics were used in the feed during the entire experiment. The composition and nutritional value of the basal diets are presented in Table 1.

**Table 1:** Raw material composition (%) and nutrition value of experimental feeds

Components (%)	Starter (day 1–21)	Grower (day 22–35)	Finisher (day 36–42)
Maize	24.44	40.00	40.00
Wheat	42.99	27.84	28.84
Soybean extraction meal*	25.0	24.97	22.87
Soy oil	2.50	3.69	3.98
1-Ca phosphate	0.90	0.90	0.81
Feed lime	1.40	1.13	1.09
Acidic sodium carbonate	0.08	0.08	0.08
NaCl	0.29	0.25	0.26
Vit.-min. prefix (no Zn)	0.50^a^	0.50^b^	0.50^c^
Protein and fat concentrate**	1.00	–	1.00
dl-methionine 99%	0.30	0.23	0.23
l-lysine HCl	0.42	0.28	0.27
l-threonine 99%	0.18	0.13	0.07
Nutrient value of 1 kg of mixture			
ME (MJ/kg)	12.7	13.1	13.2
^d^Total protein (%)	21.7	20.2	19.6
^d^Raw fiber (%)	2.41	2.32	2.31
^d^Raw fat (%)	4.52	5.28	5.64
^d^Lysine (%)	1.28	1.14	1.10
^d^Meth + Cys (%)	0.94	0.84	0.83
^d^Ca total (%)	0.87	0.79	0.76
^d^P total (%)	0.67	0.66	0.64
^e^Available P (%)	0.43	0.40	0.41
^e^Ca total/available P	2.11	1.91	1.90
^e^Fe (mg)	40.22	39.92	39.68
^e^Cu (mg)	14.54	14.72	13.59

*Forty-six percent general protein in dry matter

**One kilogram of protein and fat concentrate contains: 2% raw fat, 39% raw protein, 10.8 MJ EM

^a^Content of vitamins and minerals in 1 kg of starter mixture: Mn 100 mg, J 1 mg, Se 0.15 mg, vit. A 15000 UI, vit. D_3_ 5000 UI, vit. E 75 mg, vit. K_3_ 4 mg, vit. B_1_ 3 mg, vit. B_2_ 8 mg, vit. B_6_ 5 mg, vit. B_12_ 0.016 mg, biotin 0.2 mg, folic acid 2 mg, nicotinic acid 60 mg, pantothenic acid 18 mg, choline 1800 mg

^b^Content of vitamins and minerals in 1 kg of grower mixture: Mn 100 mg, J 1 mg, Se 0.15 mg, vit. A 12000 UI, vit. D_3_ 5000 UI, vit. E 50 mg, vit. K_3_ 3 mg, vit. B_1_ 2 mg, vit. B_2_ 6 mg, vit. B_6_ 4 mg, vit. B_12_ 0.016 μg, biotin 0.2 mg, folic acid 1.75 mg, nicotinic acid 60 mg, pantothenic acid 18 mg, choline 1600 mg

^c^Content of vitamins and minerals in 1 kg of finisher mixture: Mn 100 mg, J 1 mg, Se 0.15 mg, vit. A 12000 UI, vit. D_3_ 5000 UI, vit. E 50 mg, vit. K_3_ 2 mg, vit. B_1_ 2 mg, vit. B_2_ 5 mg, vit. B_6_ 3 mg, vit. B_12_ 0.011 μg, biotin 0.05 mg, folic acid 1.5 mg, nicotinic acid 35 mg, pantothenic acid 18 mg, choline 1600 mg

^d^Analyzed values

^e^Calculated values

The total rearing period was 42 days. One hundred fifty birds were used in one experiment, and the chickens were divided into five groups: four experimental groups and one control group. Each group, i.e., the control and the groups whose diet was supplemented with ZnSO_4_, ZnSO_4_ + F, Zn-Gly, or Zn-Gly + F, comprised 30 birds. On the first day of the experiment, 10 one-day-old chicks from each group were killed by decapitation to collect blood and liver tissue. The remaining 20 birds in each group were used for further stages of the experiment. On day 20, 10 chicks each from the 4 experimental groups and the control group were killed to collect blood and liver tissue. The remaining 10 birds in each experimental group continued to receive zinc additives, as described previously, until day 42 of the experiment. On day 42, the remaining 10 birds from all groups were killed to collect blood and liver tissue. The experiment was replicated three times. A total of 450 Ross 308 broiler chickens were used in the experiment in triplicate.

The birds in the control group (group 1) received a balanced compound feed in accordance with requirements for Ross 308 chickens, as well as a mineral and vitamin premix without zinc. In experimental group 2, zinc was added in inorganic form (ZnSO_4_), and for experimental group 3, zinc was added in the form of a phytase enzyme supplement (ZnSO_4_ + F). Experimental group 4 had zinc provided in organic form, in combination with glycine (Zn-Gly). Group 5 had zinc in combination with glycine and additionally received a phytase supplement (Zn-Gly + F). Zinc was introduced into the mineral and vitamin premix, which contained no zinc. The zinc requirement in the feed mixtures, based on the dietary recommendations for Ross 308 broiler chickens (Aviagen, Broiler Ross Nutrition Supplement, 2013, www.aviagen.com), was 100 mg/kg Zn, which did not include the content of the element in the feed components. The concentration of Zn in the water provided to the chicks during the experiment was 0.299 mg/l. The content of Zn in the experimental diets is presented in Table 2. The Zn content in the feed samples was determined using the AAS flame technique in a Unicam 939 (AA Spectrometer Unicam, Shimadzu Corp., Tokyo, Japan) apparatus, after ashing at 550 °C, according to AOAC methods [43].

**Table 2:** Analyzed Zn concentrations in diets for broilers (as-fed basis)

Zn source	Added Zn (mg/kg)	Analyzed Zn (mg/kg)		
		Starter	Grower	Finisher
Control^a^	0	23.10	21.67	21.74
ZnSO_4_^b^	100	124.90	122.15	125.90
Zn-Gly^c^	100	125.90	123.71	126.42

Values based on triplicate determinations of diet samples

^a^Control negative (corn-wheat and soybean meal control diet with no Zn supplementation)

^b^Experimental diet with 100 mg of Zn as zinc sulfate and zinc sulfate with phytase supplement/kg of diet

^c^Experimental diet with 100 mg of Zn as glycine chelate and glycine chelate with phytase supplement/kg of diet

For all experimental groups, 100 mg/kg of zinc were added to the feed. Phytase was added to the mixtures in the amount of 500 phytase activity units (FTU)/kg (RONOZYME® HiPhos, DSM Nutritional Products Sp. z o.o., Mszczonów, Poland). Glystar Forte chelate (ARKOP Sp. z o.o., Bukowno, Poland), containing 15% Zn, was used in the experiment.

All procedures used in the research were approved by the Local Ethics Committee for Animal Testing at the University of Life Sciences in Lublin, Poland (Resolution No. 37/2011 of 17 May 2011).

### Clinical Signs and Growth Performance in the Animals

Throughout the experiment, the birds were under clinical observation, with special attention paid to the activity of the birds, their appetite, respiratory symptoms, and the occurrence of digestive disorders in the form of diarrhea. In all groups, feces were tested on days 20 and 42 for the presence of *Eimeria* spp. oocysts by the flotation method, using a saturated NaCl solution, as described by Ryley and Ryley [44]. The health status of the birds was evaluated by determining clinical parameters, anatomopathological changes in dead birds, and the mortality rate (Table 3).

**Table 3:** Evaluation of health parameters

	Mortality rate (%)	Clinical symptoms in birds during the experiment	Anatomopathological changes in dead birds
Control group	2.22% (2 birds)	Diarrhea lasting 2 days, remitting spontaneously	None
ZnSO_4_	8.88% (8 birds)	Diarrhea lasting 2–4 days, remitting spontaneously	Intestinal hyperemia, petechiae in the mucosa of the small intestine
ZnSO_4_-F	10.0% (9 birds)	Diarrhea lasting 2–4 days, remitting spontaneously	Intestinal hyperemia, petechiae in the mucosa of the small intestine
Zn-Gly	1.11% (1 birds)	None	None
Zn-Gly-F	3.33% (3 birds)	None	Isolated pinpoint petechiae in the mucosa of the small intestine

During the experiment, the all chicks were weighed and their initial and final weights were calculated. The average daily gain, the feed intake (FI), and the feed conversion ratio (FCR) were determined in all birds in all group (Table 4). During the experiment, dead birds were recorded and weighed. Average daily gain (ADG) was calculated by dividing average body weight gain (average final body weight subtracted average initial body weight) by the days including weight gain of any dead birds. FCR was calculated by dividing average feed intake by average weight gain, including weight gain of any dead birds.

**Table 4:** Effect of supplementation with inorganic and organic forms of zinc on growth performance

	Initial weight (g)	Final weight (g)	ADG (g)	FI (g/bird)	FCR (g/g)
Control	44.1 ± 0.54	2226.4 ± 12.84	56.39 ± 4.23	4078 ± 179.54	1.81 ± 0.189
ZnSO_4_	43.7 ± 0.24	2126.5 ± 11.92^C^	51.47 ± 1.22^C^	3511 ± 143.03^C^	1.73 ± 0.169^C^
ZnSO_4_-F	44.2 ± 0.32	2155.5 ± 11.37^C^	51.19 ± 3.16^C^	3621 ± 172.03^C^	1.76 ± 0.168^C^
Zn-Gly	43.8 ± 0.41	2329.4 ± 8.85^AC^	56.12 ± 3.21^A^	3684 ± 161.57^AC^	1.87 ± 0.156^A^
Zn-Gly-F	43.6 ± 0.28	2450.8 ± 9.64^BC^	57.31 ± 2.18^B^	4322.4 ± 136.09^BC^	1.65 ± 0.120^BC^

Values are expressed as the mean and standard deviation (*α*^+^/− SD)

*ADG* average daily gain, *FI* feed intake, *FCR* feed conversion ratio

^A^Statistically significant differences (*p* < 0.05) between the Zn-Gly and the ZnSO_4_ group

^B^Statistically significant differences (*p* < 0.05) between the Zn-Gly-F and the ZnSO_4_-F group

^C^Statistically significant differences (*p* < 0.05) between the control group and the experimental group, assessed by the Kruskal-Wallis test and the median test

### Blood Samples

The test material consisted of 2 ml samples of peripheral blood taken from the wing vein of 1-day-old chicks killed by decapitation. The blood samples were collected in sterile vacuum tubes containing a clot activator and serum separator (Vacuette, Medlab Products, Raszyn, Poland). Blood samples were collected before the start of the study (day 0), on day 20 of rearing, and on day 42, after rearing was completed. At each stage of the experiment, 10 blood samples were collected from each group. The samples were transported to the laboratory at + 4 to + 8 °C within 1 h. Serum was obtained by centrifuging the blood at room temperature (20–22 °C) for 15 min at 4000×*g*. The serum were apportioned and stored at − 80 °C for further analysis.

### Assay of CRP, Hp, α-1-AGP, SAA, and TRF in Chicken Serum

ELISA kits specific for chicken CRP (C-reactive protein), Hp (haptoglobin), α-1-AGP (alpha-1-acid glycoprotein), SAA (serum amyloid A), and TRF (transferring) (Wuhan Fine Biotech Co., Ltd., East Lake High-tech Development District, Wuhan, Hubei Province, China) were used to determine the acute phase proteins levels in the serum. All procedures were performed according to the manufacturer’s instructions.

### Assay of CRP, Hp, α-1-AGP, SAA, and TRF in Liver Homogenates

Birds were killed on days 1, 20, and 42 of the experiment. The livers were immediately excised, rinsed of blood, and homogenized using a mechanical homogenizer in PBS that contained 0.05% sodium azide and 0.5% Triton 100× (pH 7.4). Liver samples were then sonicated for 10 min. All solid particles were removed by centrifugation of the homogenates at 12,000×*g* for 10 min. Dichloromethane (0.4 ml) was added to 1 ml of supernatant to eliminate all lipid contaminants. This process was followed by a second centrifugation for 10 min at 12,000×*g*, after which the supernatant was removed and immediately assayed. The CRP ELISA kit, Hp ELISA kit, α-1-AGP ELISA kit, SAA ELISA kit, and TRF ELISA kit were obtained from Wuhan Fine Biotech Co., Ltd. (East Lake High-tech Development District, Wuhan, Hubei Province, China). The ELISA experiments were performed according to the manufacturer’s specifications. The APP content was expressed as nanograms per milliliters of total protein.

### Determination of Zn Concentration in Chicken Liver Tissue

The Zn concentration in liver tissue was conducted using a method described by Shelton and Southern [45] and Hill et al. [46] (Table 5). Zn was separately determined in each liver sample taken from each of the birds. The liver samples from all birds from each group were collected and stored at − 20 °C until testing. Samples were dried at 100 °C for 24 h and ashed for 10 h at 550 °C. The ashed samples were dissolved in nitric acid-perchloric acid mixture (1:1) and diluted with deionized water for analysis of minerals. Contents of Zn were measured with flame atomic absorption spectrophotometry (AA-6300, Shimadzu Corp., Tokyo, Japan).

**Table 5:** Effects of zinc sulfate and zinc glycine chelate on Zn content in liver of broiler chickens

	Day	Control group	ZnSO_4_	ZnSO_4_-F	Zn-Gly	Zn-Gly-F
Liver (Zn mg/kg)	20	20.32 ± 1.29	22.83 ± 1.97	23.21 ± 2.18	26.11 ± 2.21*^A^	27.31 ± 3.11*^B^
	42	53.21 ± 4.28	55.24 ± 5.32	54.82 ± 5.44	66.18 ± 7.66*^A^	69.81 ± 8.92*^B^

Values are expressed as mean and standard deviation (*α*^+^/− SD). Asterisks indicate significant increase in the parameter between experimental groups and the control on each testing day (*p < 0.05)

^A^Statistically significant differences (*p* < 0.05) between the Fe-Gly and the FeSO_4_ group

^B^Statistically significant differences (*p* < 0.05) between the Fe-Gly-F and the FeSO_4_-F group assessed by the Kruskal-Wallis test and the median test

### Statistical Analysis

The results were analyzed statistically using Statistica 10.0 PL (StatSoft, Krakow, Poland). The analysis included the arithmetic mean and standard deviation (*α* ± SD). The significance of differences between means obtained for the control and experimental groups of animals and between sampling times was assessed by the Kruskal-Wallis and median tests, and *p* values of less than 0.05 were considered to indicate statistical significance (Tables 6 and 7). The differences in means between the results obtained for ZnSO_4_ and Zn-Gly, between ZnSO_4_-F and Zn-Gly-F, and between sampling times were assessed by the Kruskal-Wallis and median tests. *p* values for statistically significant differences are shown in Tables 6 and 7.

**Table 6:** Comparison of the concentration of acute phase proteins in chicken serum

Parameter	Day	Control group	ZnSO_4_	ZnSO_4_-F	Zn-Gly	Zn-Gly-F
SAA	0	80.19 ± 20.10	89.01 ± 7.08	84.31 ± 16.74	81.99 ± 13.92	81.95 ± 7.28
	20	146.76 ± 14.60	205.37 ± 14.53*^a^	209.97 ± 18.34*^a^	146.62 ± 12.39^a^	106.56 ± 6.37*^B^ *p* = 0.0002
	42	203.04 ± 30.45	306.63 ± 34.45*^bc^	210.93 ± 69.20^b^	191.36 ± 45.34^bc^	103.91 ± 19.03*^B^ *p* = 0.0001
CRP	0	0.87 ± 0.06	0.90 ± 0.04	0.88 ± 0.07	0.89 ± 0.05	0.88 ± 0.07
	20	1.31 ± 0.10	1.61 ± 0.17*^a^	1.14 ± 0.05	1.11 ± 0.08^A^ *p* = 0.001	1.22 ± 0.13
	42	1.89 ± 0.44	2.97 ± 0.53*^bc^	1.12 ± 0.21*	1.46 ± 0.28*^bA^ *p* = 0.014	2.06 ± 0.17*^bc^
α-1-AGP	0	10.64 ± 0.67	10.66 ± 0.96	10.64 ± 0.38	10.57 ± 0.73	10.32 ± 0.28
	20	12.07 ± 2.07	38.09 ± 4.37*^a^	25.10 ± 3.51*^a^	16.65 ± 3.28*^aA^ *p* = 0.003	23.06 ± 3.01*^a^
	42	13.79 ± 3.62	69.31 ± 6.36*^bc^	50.58 ± 14.70*^bc^	22.18 ± 3.45*^bA^ *p* = 0.006	32.28 ± 6.21*^bB^ *p* = 0.004
TRF	0	0.81 ± 0.13	0.74 ± 0.26	0.79 ± 0.19	0.87 ± 0.06	0.88 ± 0.07
	20	1.04 ± 0.07	1.80 ± 0.35*^a^	2.02 ± 0.35*^a^	1.31 ± 0.13	1.54 ± 0.11
	42	1.39 ± 0.63	1.97 ± 0.16*^b^	2.07 ± 0.40*^b^	1.51 ± 0.16^b^	4.62 ± 0.54*^bcB^ *p* = 0.002
Hp	0	0.10 ± 0.01	0.10 ± 0.01	0.09 ± 0.01	0.09 ± 0.01	0.10 ± 0.01
	20	0.21 ± 0.02	0.39 ± 0.02*	0.63 ± 0.04*^a^	0.30 ± 0.01*	0.36 ± 0.04*^B^ *p* = 0.001
	42	0.53 ± 0.09	0.61 ± 0.05^bc^	0.67 ± 0.17*^b^	0.52 ± 0.02^bc^	0.52 ± 0.06^bc^

Values are expressed as mean and standard deviation (*α* ± SD). Asterisks indicate a significant increase in the parameter between experimental groups and the control on each testing day (*p < 0.05)

^a^Statistically significant differences (*p* < 0.05) within groups between day 0 and day 20

^b^Statistically significant differences (*p* < 0.05) within groups between day 0 and day 42

^c^Statistically significant differences (*p* < 0.05) within groups between day 20 and day 42

^A^Statistically significant difference between the group of birds receiving Zn-Gly in their feed and the group receiving ZnSO_4_

^B^Statistically significant difference between the group of birds receiving Zn-Gly-F in their feed and the group receiving ZnSO_4_-F assessed by the Kruskal-Wallis test and the median test

**Table 7:** Comparison of the concentration of acute phase proteins in chicken liver tissue

Parameter	Day		ZnSO_4_	ZnSO_4_-F	Zn-Gly	Zn-Gly-F
SAA	0	100.49 ± 7.14	99.73 ± 9.43	101.90 ± 6.37	102.73 ± 7.61	100.46 ± 4.39
	20	110.21 ± 8.35	159.40 ± 8.61*^a^	190.55 ± 6.84*^a^	117.15 ± 8.81	118.28 ± 5.27^B^ *p* = 0.01
	42	150.80 ± 4.02	209.68 ± 7.01*^bc^	281.03 ± 19.68*^bc^	194.73 ± 4.89*^bc^	157.41 ± 4.48^bcB^ *p* = 0.002
CRP	0	0.69 ± 0.09	0.79 ± 0.15	0.75 ± 0.09	0.84 ± 0.08	0.83 ± 0.08
	20	1.19 ± 0.07	1.96 ± 0.17*^a^	1.68 ± 0.28*^a^	1.30 ± 0.21^A^ *p* = 0.03	1.37 ± 0.20
	42	2.11 ± 0.06	3.29 ± 0.46*^bc^	2.29 ± 0.22^bc^	1.61 ± 0.04*^bA^ *p* = 0.00002	1.81 ± 0.07*^b^
α-1-AGP	0	143.08 ± 26.88	135.46 ± 13.10	139.05 ± 20.28	135.85 ± 16.06	138.93 ± 11.43
	20	224.89 ± 34.21	178.18 ± 11.25	264.79 ± 21.56^a^	316.63 ± 21.31*^aA^ *p* = 0.0004	318.89 ± 18.31*^a^
	42	334.73 ± 34.17	248.58 ± 23.17*^b^	316.66 ± 15.68^b^	394.76 ± 11.13*^bA^ *p* = 0.005	468.93 ± 26.45*^bcB^ *p* = 0.02
TRF	0	1.08 ± 0.13	1.04 ± 0.11	1.01 ± 0.06	1.02 ± 0.06	1.02 ± 0.10
	20	1.31 ± 0.25	1.76 ± 0.12*^a^	1.97 ± 0.31*^a^	1.50 ± 0.20	4.22 ± 0.35*^aB^ *p* = 0.003
	42	3.27 ± 0.29	3.47 ± 0.24^bc^	3.83 ± 0.19^bc^	3.49 ± 0.25^bc^	8.75 ± 0.37*^bcB^ *p* = 0.03
Hp	0	9.45 ± 0.58	9.47 ± 0.51	9.58 ± 0.64	9.40 ± 0.94	9.17 ± 0.63
	20	11.96 ± 0.92	17.69 ± 1.35*	45.53 ± 3.86*	20.44 ± 0.58	149.85 ± 16.12*^B^ *p* = 0.004
	42	15.69 ± 1.27	23.28 ± 1.60*^b^	92.98 ± 3.33*^bc^	36.07 ± 2.91*^bcA^ *p* = 0.0003	329.96 ± 8.56*^bcB^ *p* = 0.02

Values are expressed as mean and standard deviation (*α* ± SD). Asterisks indicate a significant increase in the parameter between experimental groups and the control on each testing day (*p < 0.05)

^a^Statistically significant differences (*p* < 0.05) within groups between day 0 and day 20

^b^Statistically significant differences (*p* < 0.05) within groups between day 0 and day 42

^c^Statistically significant differences (*p* < 0.05) within groups between day 20 and day 42

^A^Statistically significant difference between the group of birds receiving Zn-Gly in their feed and the group receiving ZnSO_4_

^B^Statistically significant difference between the group of birds receiving Zn-Gly-F in their feed and the group receiving ZnSO_4_-F assessed by the Kruskal-Wallis test and the median test

## Results

### Clinical Signs and Growth Performance in the Animals

During the experiment, no gastrointestinal disorders or any other symptoms were observed in the birds. The birds also tested negative for *Eimeria* spp.

Evaluation of health parameters (Table 3) showed that the mortality rate of the birds in the ZnSO_4_-F and ZnSO_4_ groups was 10 and 8.88%, respectively, and was higher than in the control group and in the ZnGly and ZnGly-F groups. During the experiment, birds in the control group and in the groups supplemented with ZnSO_4_ and ZnSO_4_-F showed diarrhea symptoms lasting 2 and 2–4 days, respectively. The birds in the groups supplemented with ZnGly and ZnGly-F showed no pathological clinical symptoms. There were no anatomopathological changes in the dead birds from the control group or from the group supplemented with ZnGly. The anatomopathological examination of the dead birds in the groups supplemented with ZnSO_4_ and ZnSO_4_-F showed intestinal hyperemia and petechiae in the mucosa of the small intestine, while in the birds from the group supplemented with ZnGly-F, isolated pinpoint petechiae in the mucosa of the small intestine were observed. Detailed data are shown in Table 3.

A detailed analysis of growth performance showed that in all experimental groups, the final weight of the birds was statistically significantly higher than in the control group. However, the final weight of the birds in the group supplemented with ZnGly was statistically significantly higher than that of the birds in the ZnSO_4_ group. This parameter was also statistically significantly higher (*p* < 0.05) in the group of birds supplemented with ZnGly-F than in the birds in the ZnSO_4_-F group. Compared to the control group, statistically significantly higher average daily gain (AGD) and FI were observed in the groups supplemented with ZnSO_4_ and ZnSO_4_-F. It was also observed that the average daily gain and feed intake were statistically significantly higher (*p* < 0.05) in the ZnGly group than in the ZnSO_4_ group, as well as in the ZnGly-F group as compared to the ZnSO_4_-F group. The FCR was statistically significantly higher (*p* < 0.05) in the groups supplemented with ZnSO_4_, ZnSO_4_-F, and ZnGly-F than in the control group. The feed conversion ratio was also statistically significantly higher in the ZnGly group as compared to the ZnSO_4_ group and in the ZnGly-F group as compared to the ZnSO_4_-F group. Detailed data are provided in Table 4.

### Effects of Zinc Sulfate and Zinc Glycine Chelate on Zn Concentration in Chicken Liver Tissue

A significant increase in the Zn content between groups Zn-Gly and ZnGly-F and the control group was observed on 20 and 42 days of the study. A statistically significantly higher Zn concentration between the Zn-Gly and ZnSO_4_ group was observed on 20 and 42 days of the study. A statistically significantly higher Zn concentration between the Zn-Gly-F and the ZnSO_4_-F group was observed on 20 and 42 days of the study. Detailed data is presented in Table 5.

### Assay of CRP, Hp, α-1-AGP, SAA, and TRF in Chicken

The serum concentration of CRP was increased (*p* < 0.05) on days 20 and 42 in the group supplemented with ZnSO_4_ as compared to the control group, and the concentration of CRP was also higher on day 42 in the group supplemented with Zn-Gly-F than in the control group (*p* < 0.05). On day 42 of the study, the ZnSO_4_-F and Zn-Gly groups had lower CRP concentrations than the control group. The concentration of CRP was higher in the ZnSO_4_ group on days 20 and 42 compared to the values obtained on day 0. Also, in the control group and the groups supplemented with zinc in organic form, the concentration of CRP was higher on day 42 than on day 0. The CRP concentration differed between days 20 and 42 only in two groups: those supplemented with ZnSO_4_ and Zn-Gly-F (Table 6). A statistically significant increase in the serum concentration of CRP was observed in group ZnSO_4_ as compared to the Zn-Gly group on days 20 (*p* = 0.001) and 42 (*p* = 0.014) of the study. Detailed data are presented in Table 6.

The concentration of CRP in the liver homogenates is also shown in Table 7. A statistically significant increase (*p* < 0.05) in the concentration of CRP was observed on days 20 and 42 in the ZnSO_4_ group as compared to the control group. A similar increase was also observed on day 20 in the ZnSO_4_-F group as compared to the control group. In the Zn-Gly and Zn-Gly-F groups, the concentration of CRP in the liver homogenates was lower than in the control group. A statistically significant increase (*p* < 0.05) in the concentration of CRP was observed on day 20 in the groups supplemented with inorganic zinc as compared to the CRP concentration measured on day 0. Similarly, the concentration of CRP was increased on day 42 in the control group and all other experimental groups when compared to the CRP concentration on day 0. In the control group and in the groups supplemented with inorganic zinc, a statistically significant increase (*p* < 0.05) in CRP concentration was observed between days 20 and 42. Comparison of the CRP concentration in the liver homogenates between the supplemented groups showed a statistically significant increase in this parameter in the ZnSO_4_ group as compared to the Zn-Gly group on day 20 (*p* = 0.03) and day 42 (*p* = 0.00002) of the study.

Table 6 shows the expression of Hp in the chicken serum. Differences in the concentration of Hp were observed on day 20 in all experimental groups compared to the control group (*p* < 0.05), and differences in the Hp concentration were also observed on day 42 for the group supplemented with ZnSO_4_-F compared to the control group. Compared to day 0 of the experiment, a significantly higher concentration of Hp was observed on day 20 in the ZnSO_4_-F group. On day 42 of the study, the concentration of Hp increased as compared to day 0 in the experimental and control groups (*p* < 0.05). Statistically significant differences in the concentration of Hp also occurred in the control, ZnSO_4_, Zn-Gly, and Zn-Gly-F groups between days 20 and 42. Comparison of the serum concentration of Hp between the supplemented groups showed a statistically significant increase (*p* = 0.001) in this parameter in the ZnSO_4_-F group as compared to the Zn-Gly-F group on day 20. Detailed data are presented in Table 6.

The concentration of Hp in the liver homogenates was higher on day 42 in all experimental groups than in the control group (*p* < 0.05), but on day 20, such increases in the Hp concentration were observed only in the ZnSO_4_-F and Zn-Gly-F groups as compared to the control. The experimental groups had a significantly increased (*p* < 0.05) concentration of Hp on day 42 compared to day 0. Statistically significant differences in the concentration of Hp between days 20 and 42 occurred in the ZnSO_4_-F, Zn-Gly, and Zn-Gly-F groups (Table 7).

Comparison of the Hp concentration in the liver homogenates between the supplemented groups showed a statistically significant increase (*p* = 0.0003) in this parameter in the Zn-Gly group as compared to the Zn-SO_4_ group on day 42 of the study. This parameter was also statistically significantly higher in the Zn-Gly-F group than in the ZnSO_4_-F group on days 20 (*p* = 0.004) and 42 (*p* = 0.02). Detailed data are presented in Table 7.

The serum concentrations of α-1-AGP on day 20 increased in all the experimental groups when compared to the control group (*p* < 0.05). Also, a significant increase (*p* < 0.05) in the concentration α-1-AGP was observed on days 42 in all experimental groups when compared to day 0 values. Conversely, the increase in the concentration of α-1-AGP between days 20 and 42 was observed only in the groups supplemented with inorganic zinc. Comparison of the serum concentration of α-1-AGP between the supplemented groups showed a statistically significant increase in this parameter in the ZnSO_4_ group as compared to the Zn-Gly group on days 20 (*p* = 0.003) and 42 (*p* = 0.006) of the study. This parameter was also significantly higher in the ZnSO_4_-F group than in the Zn-Gly-F group on day 42 (*p* = 0.004). Detailed data are presented in Table 6.

Table 7 also shows the concentration of α-1-AGP in the liver homogenates. An increase in the concentration of α-1-AGP was observed on day 20 in the Zn-Gly and Zn-Gly-F groups when compared to the control group, and an increase in α-1-AGP was also noted on day 42 in the ZnSO_4_, Zn-Gly, and Zn-Gly-F groups as compared to the control group. A statistically significant increase (*p* < 0.05) in the α-1-AGP concentration was observed on day 20 as compared to day 0 in the control group, the ZnSO_4_-F group, and the groups supplemented with organic forms of zinc. On day 42, the concentration of α-1-AGP was higher in all groups when compared to day 0. A statistically significant increase (*p* < 0.05) in the concentration of α-1-AGP between days 20 and 42 was observed in the control group and in the group supplemented with Zn-Gly-F (Table 7). Comparison of the α-1-AGP concentration in the liver homogenates between the supplemented groups showed a statistically significant increase in this parameter in the Zn-Gly group as compared to the ZnSO_4_ group on days 20 (*p* = 0.0004) and 42 (*p* = 0.005) of the study. This parameter was also statistically significantly higher in the Zn-Gly-F group than in the ZnSO_4_-F group on day 42 (*p* = 0.02). Detailed data are presented in Table 7.

The concentrations of SAA in the serum of the birds are shown in Table 6. There were statistically significant differences between the concentrations of SAA in the ZnSO_4_ and Zn-Gly-F groups; these differences were observed on both days 20 and 42 of the study. In the group supplemented with Zn-Gly-F, the concentration of SAA on days 20 and 42 was lower than in the control. A significant increase (*p* < 0.05) in the SAA concentration was observed in the ZnSO_4_-F group, but only on day 20. In all groups except for the Zn-Gly-F group, a statistically significant increase (*p* < 0.05) in the level of SAA was observed on days 20 and 42 as compared to day 0. Differences in SAA concentrations between days 20 and 42 were observed in the control group and in the ZnSO_4_ and Zn-Gly groups (*p* < 0.05). Comparison of the serum SAA concentration between the supplemented groups showed a statistically significant increase in this parameter in the ZnSO_4_-F group as compared to the Zn-Gly-F group on days 20 (*p* = 0.0002) and 42 (*p* = 0.0001). Detailed data are presented in Table 6.

Compared to the control group, the SAA levels in the liver homogenates showed statistically significant differences (*p* < 0.05) on both days of the study in the groups supplemented with inorganic zinc on days 20 and 42. However, the SAA concentration differed in the Zn-Gly group only on day 42. Statistically significant differences (*p* < 0.05) in the SAA concentrations occurred on days 20 and 42 in the groups supplemented with inorganic zinc when compared to the values measured on day 0. Statistically significant differences (*p* < 0.05) in SAA concentrations were found on day 42 in the control group and in the groups supplemented with organic zinc when compared to the values measured on day 0. In all experimental groups and in the control, the concentration of SAA increased between days 20 and 42 (Table 7). Comparison of the SAA concentration in the liver homogenates between the supplemented groups showed a statistically significant increase in this parameter in the ZnSO_4_-F group as compared to the Zn-Gly-F group on days 20 (*p* = 0.01) and 42 (*p* = 0.002). Detailed data are presented in Table 7.

The concentrations of TRF in the chicken serum are shown in Table 6. The concentration of TRF increased on days 20 and 42 in the groups supplemented with inorganic zinc as compared to the control group on days 20 and 42 (*p* < 0.05). Also, the concentration of TRF increased on day 42 in the Zn-Gly-F group as compared to the control group on day 42 (*p* < 0.05). In the groups supplemented with inorganic zinc, the concentration of TRF increased on days 20 and 42 as compared to day 0. In the groups supplemented with organic zinc, the concentration of TRF increased on day 42 as compared to day 0. Significant differences (*p* < 0.05) in TRF concentrations between days 20 and 42 were observed only in the group supplemented with Zn-Gly-F. Comparison of the serum concentration of TRF between the supplemented groups showed a statistically significant increase (*p* = 0.002) in this parameter in the Zn-Gly-F group as compared to the ZSO_4_-F group on day 20. Detailed data are presented in Table 6.

Table 7 also shows the concentration of TRF in the chicken liver homogenates. On day 20, the TRF concentration increased in the groups supplemented with inorganic zinc compared to the control group (*p* < 0.05). On day 42, the TRF concentration increased in the Zn-Gly-F group compared to the control group (*p* < 0.05). Also on day 42, the TRF concentration was increased in all groups compared to day 0 (*p* < 0.05). On day 20, the TRF concentration was increased in the groups supplemented with inorganic zinc compared to day 0 (*p* < 0.05). In all the experimental groups and in the control group, there was an increase in the concentration of TRF between days 20 and 42. Comparison of the TRF concentration of TRF in the liver homogenates between the supplemented groups showed a statistically significant increase in this parameter in the Zn-Gly-F group as compared to the ZnSO_4_-F group on days 20 (*p* = 0.003) and 42 (*p* = 0.03). Detailed data are presented in Table 7.

## Discussion

Numerous studies have found that zinc is best assimilated in poultry in the form of chelates, including glycine chelates [10, 47]. The use of this type of zinc compound as a feed additive makes it possible to administer smaller doses and to ensure proper assimilation of the zinc by the birds, leading to improved performance parameters. Nevertheless, an excessive supply of zinc glycine chelates together with phytase may stimulate cellular immune mechanisms by increasing the proliferation of T lymphocytes and immune reactivity [6]. Our previous work indicates that an excessive supply of zinc leads to increased concentrations of IL-2, TNF-α, and IL-10, which regulate homeostasis in the body and take part in the development of the local immune process within the intestines [6]. Our research has also shown that the use of zinc sulfate can lead to a local inflammatory response in the intestines, as evidenced by the high concentration of pro-inflammatory cytokines in the birds receiving zinc in this form [6]. These cytokines initiate and modulate the *acute phase response* (APR) in poultry, manifested as synthesis of numerous proteins, which can aggravate or reduce inflammation. The severity of the inflammatory response is generally in inverse proportion to the serum concentration of zinc [48]. During the development of local or systemic inflammation, zinc has been shown to be redistributed from the blood to other tissues, especially the liver, where it can increase synthesis of APP [35]. The redistribution of zinc from the blood to other tissues should be kept in mind when poultry feed is supplemented with various forms of zinc, because, although it is an essential element, it can lead to disturbances of homeostasis [9, 24, 25].

The liver plays an important role in the metabolism of zinc as the storage site for this element and the site of synthesis of various proteins involving the participation of zinc, such as APP [24, 25, 49]. The results of our experiment showed that feed supplementation with Zn-Gly and Zn-Gly-F caused an increase (*p* < 0.05) in Zn concentration in the liver tissue on days 20 and 42 of the study compared to the control group. The highest Zn concentration in the hepatic tissue was observed in birds receiving Zn-Gly-F. Similar observations were made by Tronina et al. [50], who showed that supplementation of broiler chickens with Zn-glycine resulted in increased Zn concentrations in the liver and other tissues. Zhang et al. [51] showed that Zn-Gly supplementation increases the concentration of zinc in eggs and in the liver of developing embryos and day-old chicks, which affects hatching and rearing parameters in birds. Similarly, Tang et al. [49] showed that the use of clinoptilolite as a zinc carrier increases the concentration of this element in the liver, pancreas, and bones of birds, which is linked to its effect on metallothionein mRNA and transporters mediating the intestinal adsorption of Zn. In our study, the higher zinc concentration in the liver of birds receiving Zn-Gly and Zn-Gly-F indicates better bioavailability of the microelement administered in organic form compared to inorganic forms. This is confirmed by research by Baker, 1995 [52] as well as the opinion of the Scientific Committee on Animal Nutrition referring to the use of zinc as an animal feed supplement [53]. Different observations were made by El-Husseiny et al. [54], who showed that the use of chelates and inorganic compounds containing Zn, Mn, and Cu in poultry diets does not affect the concentration of these elements in the liver of broiler chickens, whereas their content in feed exceeding the NRC recommendations results in a significant increase in the concentration of these minerals in the liver tissue. In our experiment, the increase in Zn concentration in the liver tissue of birds supplemented with zinc glycine chelate, in conjunction with the concentration of APP and the clinical evaluation, indicates that the organic form of zinc more effectively protects the body against inflammatory processes. However, Zn metabolism is known to be dynamic, and the redistribution of this element between tissues and body fluids proceeds continuously regardless of the supply of the element in the diet [55]. Long-term intake of highly bioavailable zinc with feed may also lead to lower absorption of other elements and metabolic disorders [55, 56].

In most animal species, including poultry, the main APP is SAA [57]. It is a first-line protein that has an immunomodulatory or inhibitory effect on pro-inflammatory agents that cause tissue damage [29, 58]. Studies have demonstrated that SAA can be used to monitor the course of diseases in poultry [20, 26]. Nazifi et al. [31, 59] showed an increased concentration of SAA in chickens infected with infectious bursal disease virus (IBDV) and infectious bronchitis (IB), and Upragarin et al. [26] reported an increase in SAA in the case of bacterial infections induced by *Staphylococcus aureus*. Furthermore, Chamanza et al. [20, 21] and Sevimli et al. [23] showed that SAA is a fast-reacting APP in chickens and that its concentration is increased by the activity of pro-inflammatory cytokines, particularly IL-1β, IL-6, and TNF-α.

As a feed additive, zinc affects nutrient metabolism, leading to changes in the production of hepatic proteins, including the APPs, which mediate the absorption, transport, and tissue accumulation of amino acids, lipids, vitamins, and minerals [23, 60]. In our study, a higher serum concentration of SAA was noted on days 20 and 42 of the experiment in the group of chickens that received zinc sulfate, and these concentrations were statistically significantly higher than in the group of birds receiving Zn-Gly (Table 6). Similarly, higher concentrations of SAA were observed in the liver tissue on days 20 and 42 in the group of birds that received zinc sulfate with phytase, and these concentrations were also statistically significantly higher than in the group of birds receiving Zn-Gly-F (Table 7). These data indicate activation of the acute phase response (APR), probably as a result of local inflammation induced by the zinc sulfate compounds. The activation of the synthesis of APPs by pro-inflammatory cytokines is confirmed by the increased concentration of TNF-α in the birds receiving zinc sulfate [6]. The increased serum concentration of SAA in the experimental groups and the increased concentration of SAA in the liver tissue of the experimental and control groups may also be a response to colonization of the gut by saprophytic microbes [61]. These microbes colonize the intestines during the ontogenetic development of birds. It is interesting that a low SAA concentration was obtained in the serum of birds in the group of birds receiving Zn-Gly + F on both days 20 and 42 in comparison with the corresponding values for the control and other experimental groups. It is likely that the SAA synthesis in the liver of the birds in group Zn-Gly + F was proceeding properly, and the low serum concentration of SAA suggests that there was no inflammatory process taking place and that SAA was only taking part in processes maintaining homeostasis. The highest weight gains were obtained in this group of birds, as well as better intake and feed conversion (Table 4).

Reports in the literature indicate that the SAA concentration can be influenced by a variety of stress factors [18, 19, 62]. The use of various zinc compounds as feed additives can cause a stress response in birds, such as the irritating effect of zinc sulfates on the intestinal epithelium. In these conditions, homeostasis is disturbed and manifests as an increased concentration of SAA [57]. This is confirmed by the anatomopathological examination in our study, in which the dead birds from the groups that had received ZnSO_4_ and ZnSO_4_-F exhibited inflammatory changes, which can lead to damage to the intestinal barrier and to secondary infections.

Another APP occurring in poultry that is important for diagnosis and prognosis is AGP, which is synthesized and secreted by the hepatocytes [27]. AGP exhibits immunoregulatory properties and takes part in the regulation of the inflammatory response, impairing neutrophil function [38]. An increased concentration of AGP is observed in poultry during infections, particularly in the early stages of the inflammatory process [28, 63, 64]. In our study, a statistically significantly higher serum AGP concentration was noted on days 20 and 42 of the study in the group of birds receiving ZnSO_4_ and on day 42 in the group receiving ZnSO_4_-F. The high concentration of AGP in the groups receiving zinc sulfate with or without phytase may be linked to the irritating effect of zinc sulfates on the intestinal mucosa, which was shown in the anatomopathological examination of the dead birds from these groups (Table 3). This leads to local inflammation and the destruction of intestinal epithelial cells and may increase colonization of the mucosa by pathogenic microbes. On the other hand, the low concentration of AGP in the liver of the chickens in the groups receiving ZnSO_4_ and ZnSO_4_ + F may have been due to the secretion of AGP from the liver into the cardiovascular system, which takes place in the APR to local inflammatory changes in the intestines. It is worth emphasizing that the AGP concentration in the liver tissue was higher in the groups of birds that received the zinc glycine chelate. The high concentration of AGP in the liver of the birds from the groups receiving Zn-Gly and Zn-Gly + F suggests that AGP has an immunoregulatory effect, as it is known to affect the function of T lymphocytes by inhibiting their proliferation [23, 65]. The chickens receiving zinc glycine chelates had a higher percentage of T lymphocytes with CD3^+^CD4^+^, CD3^+^CD8^+^, and CD25^+^ expression, which stimulate cellular immune response mechanisms [6]. Some of these cells, however, function as regulatory lymphocytes. These results, in conjunction with the AGP concentration in the liver, suggest that these cells will inhibit inflammatory responses induced by excessive supply of zinc in the form of chelates, thereby maintaining homeostasis. This is also confirmed by the results of the anatomopathological examination, as in the groups receiving the zinc glycine chelate no intensive inflammatory processes were observed in the intestines, and the mortality rate was very low. In contrast, in the birds receiving zinc sulfate, the mortality rate was higher and we observed acute changes in the form of hyperemia and petechiae on the intestinal mucosa (Table 3). Libert et al. [36] showed in mice that AGP protects the organism against the damaging effect of TNF-α cytokines. The high concentration of TNF-α cytokines and the intensification of phagocytosis observed in our previous study in chickens receiving a zinc glycine chelate, in conjunction with the high concentration of AGP observed in the present study, indicates that these proteins interact to limit the APR by stimulating the cytotoxicity of macrophages and heterophils, which counteract infections by pathogenic microbes. This is also confirmed by the growth performance and health parameters of the chickens (Tables 3 and 4).

High concentrations of AGP in chickens are usually noted during the inflammatory processes accompanying bacterial infections, as observed by authors such as Dunkley et al. [66], who reported an AGP concentration of over 400 μg/ml in the case of infection with *Salmonella enterica* serovar *Enteritidis*. In the present study, such a high concentration of AGP was not observed in any of the birds on any of the test days, so bacterial infections can be ruled out in the experimental and control birds.

It should be emphasized that the stress response leads to glucocorticoid release, which affects the metabolic pathways in the liver and increases the expression of most of the APP genes, including the AGP gene [67]. Overexpression of the AGP gene in the liver of the chickens receiving the zinc glycine chelate may have been induced by the increased supply of available zinc, which causes an increase in the cortisol concentration [19]. The effect of non-inflammatory stress factors on the APPs has been the subject of research by numerous authors [32, 63, 68]. Their work has shown that overcrowding of birds in cages leads to increased serum concentrations of ovarian venous plasma (OVT), AGP, and ceramide (CER). Zulkifli et al. [69] reported that the serum concentration of AGP was nearly 60 ng/ml following administration of corticosterone to birds to induce a stress response. In the present study, such high values were not observed in the chickens in the control group nor in the groups receiving the zinc glycine chelate on any of the three assay days, so we rule out the effect of stress on the concentration of the APPs. Furthermore, it should be noted that all chickens in the control and experimental groups were kept in identical environmental conditions throughout the experiment. Thus, we can discount the effect of environmental conditions on our results, which therefore depend only on the type of zinc preparation used to supplement the feed and on the individual characteristics of the birds.

During inflammatory processes in birds, changes also occur in the concentrations of other proteins, including TRF, which is therefore included among the positive APPs [60, 70]. The concentration of TRF in the serum and liver has been shown to increase during the course of bacterial and viral infections, and thus, the concentration of TRF can be used as a diagnostic marker of inflammation or can determine the onset of infection in chickens [30, 33, 71]. In the present study, no significant rapid increase in the TRF concentration was shown in the serum or liver tissue, which demonstrates that the change in the concentration of this protein was linked to the type and availability of the zinc preparation. The statistically significant increase in the TRF concentration in the group of birds receiving the zinc glycine chelate together with phytase, noted on days 20 and 42 in the liver and on day 42 in the serum in comparison with the birds receiving ZnSO_4_-F, may have been caused by the irritating effect of the excessive quantity of easily assimilated zinc, which had an immunomodulatory effect on the intestinal epithelium. TRF also exerts an immunomodulatory effect, thus preventing the multiplication of bacteria, stimulates the activity of heterophils and macrophages, and facilitates tissue reconstruction and angiogenesis, thereby aiding tissue repair processes [33, 71]. Our earlier research [6] shows that supplementation of feed with zinc glycine chelates causes an increase in the percentage of phagocytic monocytes and heterophils, in the proliferative activity of CD4^+^ T lymphocytes and in the concentration of TNF-α. In conjunction with these prior results, the TRF concentration in the serum and liver showed that the zinc glycine chelates may have stimulated non-specific defense mechanisms and intensification of immunoregulatory processes in the birds. These results confirm findings from a study by Xie et al. [30] demonstrating that TRF modulates the activity of macrophages and heterophils, exerts a bactericidal and anti-inflammatory effect, and helps to maintain homeostasis. The group of chickens that received zinc sulfate or zinc sulfate with phytase showed a significant increase in the serum concentration of TRF on day 20 only in comparison with the control. Taken together with the high concentrations of the other APPs, the serum concentration of TRF suggests that long-term use of these preparations may lead to the development of local inflammatory changes, which was also evident in the anatomopathological examination of the dead birds from these groups. The development of local inflammatory changes is confirmed by the results of our earlier study [6], in which birds receiving zinc sulfates had high concentrations of pro-inflammatory cytokines IL-2 and TNF-α.

Hp is an APP with a strong anti-inflammatory effect that modulates prostaglandin synthesis and inhibits granulocyte chemotaxis and phagocytosis [24]. Hp also exhibits antioxidant properties, preventing tissue damage [72]. Seifi et al. [34] showed that in birds infected with infectious bronchitis virus (IBV), the concentration of Hp increased rapidly in the first 24 h after infection. The increase in the serum concentration of Hp in all groups on day 42 of the experiment in comparison with the control may indicate stimulation of local immune mechanisms. In our study, the statistically highest Hp concentration in the liver tissue was observed in the group receiving the zinc glycine chelate with phytase on days 20 and 42 of the study and in the group receiving the zinc glycine chelate on day 42, as compared to the groups receiving zinc sulfate with phytase and zinc sulfate. This may be linked to stimulation of protein synthesis by zinc and mobilization of immunocompetent cells of the gut-associated lymphoid tissue (GALT) to locally synthesize Hp [73].

In most animal species, an important early marker of an ongoing inflammatory process is an elevated level of the CRP [25]. An increase in the concentration of CRP is linked to cell damage caused by bacteria, viruses, fungi, and parasites within 36–48 h after exposure to the damaging stimulus. A low CRP concentration in poultry is also observed in the serum of seemingly healthy birds [22]. This low concentration of CRP is in part linked to the presence of chronic lesions in the organs and tissues, indicating that CRP can be used as a general health marker. In the present study, the statistically significantly highest serum concentration of CRP in the chickens was noted on days 20 and 42 in the group receiving zinc sulfate, which may indicates the development of a local inflammatory process induced by the irritating effect of this compound. This was also evident in the anatomopathological examination of the dead birds from this group. A high CRP concentration was also found in the group of chickens receiving the zinc glycine chelate as compared to the control. No statistically significant differences were noted between the group of birds receiving the zinc glycine chelate with phytase and the birds receiving zinc sulfate with phytase. This suggests stimulation of the enterocytes of the intestinal epithelium and the stimulation of local immune mechanisms due to excessive supply of zinc in the form of a chelate. Similarly, the statistically highest CRP concentration in the liver tissue was noted in the group of birds receiving zinc sulfate, which suggests the stimulation of hepatic synthesis of APP in response to a developing inflammatory process. In the remaining experimental groups and in the control group, the CRP concentrations were similar. Because an increase in CRP synthesis in the liver stimulates the synthesis and release of TNF-α, evaluation of the serum concentration of CRP can be used to monitor the severity of the TNF-α-dependent inflammatory response [74]. In the present study, the highest CRP concentration was found in the group of chickens receiving zinc sulfate, and we previously found no significant increase in the TNF-α concentrations in such birds [6]. These results confirm the pro-inflammatory nature of the effect of diet supplements containing zinc sulfates, which can initiate infections within the gastrointestinal tract after long-term use.

The literature indicates that the systemic and local immune response in poultry affects intestinal motility and may interfere with the absorption and metabolism of nutrients, including zinc, thus enabling the multiplication of microbes and increasing morbidity and mortality rates [9, 74]. It has also been shown that in the case of a local inflammatory response, such as one induced by irritants, the distribution of individual minerals is disturbed, which significantly impairs their function [75]. With age and ontogenetic development, differences appear in the structure and function of the intestines of birds [75], including the length of the villi and the intensity of apoptosis of enterocytes. Due to age-associated differences in the gastrointestinal tract of birds, it is important to use feed of suitable quality, with nutrients having immunostimulatory or immunosuppressive effects [75]. This necessity is associated in part with age-dependent variations in the uptake of nutrients from feed, which affects immune function. The limited availability of nutrients like zinc inhibits the development of lymphoid tissue in the digestive tract, such as the bursa of Fabricius and the lymphatic system of the intestine [3], and also affects the type of bacteria colonizing the intestines [76]. As the intestines develop, fluctuations occur in the synthesis of the APP that exhibit immunoregulatory effects [22, 25, 38]. The results of the present study showed that supplying an appropriate amount of bioavailable zinc in the form of a glycine chelate ensured the immunomodulatory effect of this element, which is essential for maintaining homeostasis. The concentrations of the selected APPs in the chickens receiving the zinc glycine chelate and in the control did not indicate that the form of zinc used in the study played a role in the APR. The results of the blood serum and liver tissue tests together with the clinical evaluation of the birds during the experiment indicated an immunomodulatory effect of the zinc glycine chelate on the body during development and suggest that this formula of zinc can be recommended as a dietary supplement for poultry.

## Conclusion

Zinc in the form of a glycine chelate added to feed had the most beneficial effect for poultry in terms of health, performance, and immunity. The increased concentrations of the APPs such as AGP and TRF following administration of zinc glycine chelates demonstrates their effect on metabolic processes in the liver and on immunocompetent cells that regulate the intensity of the immune response. The use of zinc sulfate feed additives, on the other hand, caused a local inflammatory response that manifested as increased concentrations of CRP, AGP, and SAA. Such an inflammatory response can lead to disturbances in homeostasis, damage to the intestinal barrier, secondary infections, and the development of a generalized inflammatory response. Understanding the effect of zinc sulfates and zinc glycine chelates on enterocyte function and on the local immune system of the intestinal mucosa (e.g., GALT) will require further study.
